# Prevalence and associated risk factors of hypertension and pre-hypertension among the adult population: findings from the Dubai Household Survey, 2019

**DOI:** 10.1186/s12872-022-02457-4

**Published:** 2022-01-28

**Authors:** Heba Mamdouh, Wafa K. Alnakhi, Hamid Y. Hussain, Gamal M. Ibrahim, Amal Hussein, Ibrahim Mahmoud, Fatheya Alawadi, Mohamed Hassanein, Mona Abdullatif, Kadhim AlAbady, Sabya Farooq, Nabil Sulaiman

**Affiliations:** 1 Department of Data Analysis, Research and Studies, Dubai Health Authority, Dubai, UAE; 2 Department of Family Health, High Institute of Public Health, Alexandria University, Alexandria, Egypt; 3 Mohammed Bin Rashid University of Medicine and Health Sciences, Dubai, UAE; 4 Department of Data Analysis, High Institute for Management Sciences, Belqas, Egypt; 5 Department of Family and Community Medicine and Behavioral Sciences, College of Medicine, University of Sharjah, Sharjah, UAE; 6 Department of Diabetes and Endocrinology, Dubai Hospital, Dubai Health Authority, Dubai, UAE; 7 Public Health Protection Department, Dubai Health Authority, Dubai, UAE; 8 Baker Heart and Diabetes Institute, Melbourne, VIC Australia

**Keywords:** Hypertension, Pre-hypertension, Dubai population, Socio-demographics, Behavioral risk factors

## Abstract

**Background:**

Minimal data is available on the prevalence and correlates of hypertension and prehypertension in Dubai. The study aims to measure the prevalence of hypertension and pre-hypertension and the associated socio-demographic characteristics, behavioral risk factors and comorbidities among the adult population of Dubai.

**Methods:**

This study used data from the Dubai Household Health Survey, 2019. A cross-sectional population survey based on a complex stratified cluster random design. The total eligible sample included 2530 adults (18+). Sociodemographic and behavioral factors were considered as independent covariates. The main study outcome variables, pre-hypertension and hypertension, were ordinal, with normotension as the reference group.

**Results:**

The overall prevalence of hypertension in adults was 32.5% (38.37% in males and 16.66% in females). Prehypertension was prevalent in 29.8% of adults in Dubai (28.85% in males and 32.31% in females). The multivariate logistic regression analysis revealed that age groups, gender, occupation, and high Body Mass Index were significantly associated with a higher risk of hypertension at the level of *P* < 0.05. No clear trend toward a higher correlation of hypertension was noted with the increase in age, except after the age of 50 years. Males were five- times more likely to be hypertensive than females. Participants enrolled in skilled and service works had a five times higher risk of hypertension, compared with the reference group (professionals). Obese subjects had a 5.47-times greater correlation of hypertension compared with normal-weight subjects. Physically active individuals were less likely to develop hypertension. For the correlates with prehypertension in the present analysis, skilled and service workers and those working in elementary jobs had a higher risk of prehypertension, compared with the reference group (professionals) Individuals with a status of overweight were associated with a higher prevalence of prehypertension compared with people of normal weight.

**Conclusions:**

This study showed a high prevalence of prehypertension and hypertension among adults in Dubai. Some socio-demographic and behavioral risk factors were correlated with prehypertension and hypertension among the studied population. Interventions aiming at increasing public awareness about such risk factors are essential.

## Introduction

Hypertension (HTN), defined as elevated blood pressure beyond normal ranges, is recognized as one of the major non-communicable health disorders and a public health challenge worldwide [1]. According to the World Health Organization (WHO), hypertension accounts for one out of eight mortalities worldwide [2]. Hypertension prevalence has risen over the last decades around the world. The latest estimates showed that nearly one-third of the adult world population is having hypertension (31.1%, 1.39 billion); of whom two-thirds are living in low- and middle-income countries (LMICs) [3]. A systematic analysis of population-based studies from 90 countries estimated that hypertension among adults was more prevalent in LMICs (31.5%) than in high-income countries (28.5%) [4]. In particular, the Gulf countries have become one of the most affected in the region with the rising trends of HTN in the world [5]. This is due to the rapid economic development, speedy urbanization, aging of the population, unhealthy dietary habits and lifestyle changes that occurred during the past decades within these countries [6]. Such epidemiological transition significantly contributes to changes in disease patterns and causes a growing number of undiagnosed, diagnosed and uncontrolled hypertensive individuals in many settings [7].

The term prehypertension (PHTN) has been introduced in 2003 to draw attention to the increased risks resulting from high blood pressure [8]. People with PHTN reported having a higher risk of progressing to HTN and an elevated risk of cardiovascular morbidity and mortality [2, 9, 10]. Currently, there is a rising potential for primary prevention and early detection efforts to prevent the transition from PHTN to HTN and the increase in cardiovascular risks [11].

Previous studies have identified several factors that put people at risk of developing HTN [4, 11, 12]. Some are non-modifiable factors such as age, gender, family history, and ethnicity. On the other hand, smoking, alcohol consumption, diet, physical inactivity, Body Mass Index (BMI), abdominal obesity, stress, hyperglycemia, and hypercholesterolemia are among the many modifiable correlates of hypertension [12–15].

Dubai is one of the seven Emirates that make up the United Arab Emirates (UAE) [16]. The Dubai Health Authority (DHA) is the government entity that oversees the health status of the population and ensures excellence in healthcare provision to all residents [17]. Therefore, DHA conducts Dubai Household Surveys (DHHS) on a five-year interval basis to investigate a wide range of health, social and economic issues as well as assessing for PHTN and HTN among the sampled adult population of Dubai.

Studies on the prevalence and correlates of PHTN and HTN among the Dubai population are scarce. Therefore, this study aimed to measure the prevalence of hypertension and pre-hypertension among the adult population of Dubai and to identify the associated sociodemographic characteristics, behavioural risk factors, and comorbidities.

## Methodology

### Data source and study design

This study used data from the 2019 DHHS. The DHHS is a population-based cross-sectional survey that was designed to assess the health status of the population of Dubai. The survey used a multistage stratified cluster sampling technique, based on the WHO World Health Surveys criteria. Clusters were selected based on the simple random method. The clusters were settled as primary sampling units in each stratum, each of which ranged in size from 100 to 200 households. Families were randomly reached by withdrawing a specified number of initial units in each stratum as a first stage, and a specific number of households were withdrawn from each enumeration unit in the second stage. In addition, a number of replacement families were planned to compensate for non-responses. Each participant was assigned a sampling weight, which was inversely proportional to the probability of selection. The proportion of UAE-nationals within the sample was over-sampled, and this was corrected by sample weighting. Out of the total number of adults (18+ years) accepted to participate in the survey, 2530 (include both Emirati and non-Emirati) had their blood pressure measured successfully, which identified the sample size for the current study.

In addition to socio-demographic characteristics and behavioral risk factors data collected through a pre-designed questionnaire, participants underwent selected clinical measurements, including blood pressure (BP), anthropometry, and laboratory tests to measure blood sugar levels.

### Variables ad measures

Socio-demographic variables included age, gender, marital status, nationality, education, occupation, and work status. Marital status was categorized as married, single, or others (divorced/ and widowed). Nationality was a categorical variable with four groups: UAE-Nationals, Asians, other Arabs, and Western and others. Participant’s educational attainment was classified as; ‘less than secondary’, ‘completed secondary or high diploma’, and ‘university degree or higher education’. Participants’ occupations were classified based on the international standard classification of occupations and were then re-grouped into three categories; (1) professionals, (2) skilled and service (3) elementary or unskilled occupations, and others [18]. Besides, the socio-demographics, the study also inquired about the health behavior risk factors including smoking, physical activity status and alcohol use. Adequate fruit and/or vegetable consumption was considered if the participant consumed at least five servings/day. Physical activity status was classified as “active” as per the WHO recommendations of at least 30 min of regular, moderate-intensity physical effort for at least five days a week, totaling 150 min [19]. Participants were considered as current smokers if they reported smoking any type of tobacco product during the time of the survey. Alcohol consumption was defined as any alcohol usage during the month preceding the survey.

### Outcome variables

HTN and PHTN were operationally defined according to the diagnostic cut-off points of the WHO criteria [8]. Throughout this study, the prevalence of hypertension was based on both self-reported individuals that were previously diagnosed with hypertension and the newly diagnosed during the survey (undiagnosed hypertension). A normal adult has systolic blood pressure (SBP) below 120 mmHg and diastolic blood pressure (DBP) of less than 80 mmHg and was identified as normotensive in the current analysis. Pre-hypertension (recently named as elevated BP) is diagnosed with SPB of 120–139 mmHg or DBP of 80–89 mmHg [20]. While hypertension is defined as SBP of equal to or above 140 mmHg and DBP equal to or above 90 mmHg. Participants were screened for hypertension by blood pressure measurements using calibrated sphygmomanometers. Each individual had three blood pressure measurements at two points of time after the first measurement. The mean of the three blood pressure readings was calculated and the participant was labeled as a newly diagnosed hypertensive patient if the definition was met.

Medical conditions included self-reported chronic diseases (referred to as having a chronic condition from a predefined list), having a history of high cholesterol and a history of repeated chest pains. Diabetes status was based on HbA1c test cut-off measures. Participants with HbA1c levels below 6.5% were considered non-diabetic while those with HbA1c equal to or above 6.5% were considered as diabetic, according to the WHO criteria [21]. The overall prevalence of diabetes was based on both the self-reported status and the newly diagnosed diabetes during this survey. BMI was calculated using the formula body weight (in kg)/ body height (in m)^2^. BMI values below 25 were considered normal, while values between 25 and 29.9 were classified as overweight, and BMI values of 30 and above indicated obesity as per the WHO Growth Reference [22].

### Statistical analysis

Data coding, data cleaning, and analysis have been carried out by using IBM SPSS (Version 21.0, IBM SPSS, IBM Corp, Armonk, NY, USA) and Stata (Version 15, Stata Corporation, College Station TX). Frequencies and relative frequencies were reported for categorical variables. The Chi-square test of association was used to conduct bivariate analyses and study associations between categorical variables and the outcome variables (hypertension and pre-hypertension). Following the Chi-square test analysis and in order to identify cells with significant findings, post hoc analysis was performed by calculating *P* values for the adjusted residuals. *P* values were then compared to the adjusted alpha value using the Bonferroni correction to correct for the inflated type 1 error (data is not provided in the text). Multivariate logistic regression analyses were performed to evaluate significant risk factors for prehypertension (versus normotension) and hypertension (versus normotension). The potential predictors were age, gender, BMI, occupation, smoking status, alcohol consumption and physical activity status. Odds ratios (OR) were reported to reflect the strength of association. All the statistical tests were two-sided. The level of significance was set at 5% (*P* < 0.05) and accordingly, confidence intervals (CIs) were calculated with a 95% level. Complex weighted computation was used to report the weighted prevalence of HTN and PHTN.

## Results

The total eligible sample included 2530 adults (18+ years). Table 1 shows the weighted prevalence of HTN and PHTN according to demographic characteristics. The overall prevalence of HTN was 32.5% (38.37% in males and 16.66% in females). Prehypertension was prevalent in 29.8% of adults in Dubai (28.85% in males and 32.31% in females). A clear trend toward a greater prevalence of HTN was noted as age increased; however, the prevalence of PHTN was fluctuating among the age groups. Individuals aged 60+ had the highest proportion of HTN (56.48%). Moreover, Asians reported the highest frequency of HTN (34.69%). However, those from western countries and others had the highest frequency of PHTN (45.05%) among all other nationalities. Divorced and widowed individuals had a higher prevalence of both PHTN (38.45%) and HTN (40.37%) than other marital status categories, *P *value < 0.001. Individuals who were currently working had significantly higher (44.06%) HTN, and among them skilled and service workers had the highest percentage (42.94). Individuals with less than secondary education had higher HTN (44.06%) than those with bachelor’s education and post-graduates. Using the univariate analysis, the prevalence of PHTN and HTN was significantly different within different age groups, gender, nationality groups, marital status, educational attainments, and occupations (all *P *values < 0.001). Table 2 showed the descriptive univariate analysis for medical conditions associated with PHTN and HTN among the participants. Self-reported histories of certain chronic disorders like high blood cholesterol levels, chest pain, diabetes mellitus (both self-reported and measured), and the measured BMI disorders were all significantly associated with PHTN and HTN, (*P* < 0.001). Hypertensive subjects reported other chronic diseases, high cholesterol levels, and chest pain. Among diabetics in this survey, 61.38% had HTN, and 55.85% of obese individuals had HTN.

**Table 1:** Prevalence of PHTN and HTN among Dubai population according to socio-demographic characteristics

Characteristics	Prevalence %			^*P* value
	Normotensive	PHTN	HTN	
*Age group*				
18–29	49.13	30.98	19.89	< 0.001
30–39	39.97	29.95	30.08	
40–49	33.16	28.41	38.42	
50–59	18.20	27.90	53.90	
60+	11.25	32.27	56.48	
*Gender*				
Females	51.03	32.31	16.66	< 0.001
Males	32.78	28.85	38.37	
*Marital status*				
Single	48.69	30.12	21.19	< 0.001
Married	33.71	29.37	36.91	
Divorced/separated/widowed	21.18	38.45	40.37	
*Nationality*				
UAE-Nationals	42.26	32.59	25.15	< 0.001
Other Arabs	41.98	30.98	27.03	
Asians	38.02	27.29	34.69	
Western and others	27.93	45.05	27.02	
*Education level*				
Less than secondary	29.92	26.02	44.06	< 0.001
Completed secondary and high diploma	37.16	29.45	33.40	
Bachelor degree or post-graduates	42.00	31.92	26.08	
*Occupation*				
Professionals	38.74	32.08	29.18	< 0.001
Skilled and service workers	30.28	26.78	42.94	
Elementary or unskilled workers	40.39	27.98	31.63	
*Work status*				
Not currently working	40.52	32.92	26.57	< 0.001
Working	37.19	29.22	33.59	
*Total prevalence*				
37.71	29.79	32.50		

Results are presented as weighted proportions for all the categorical variables

*HTN* hypertension, *PHTN* prehypertension

^^^χ2 test

**Table 2:** Medical conditions associated with PHTN and HTN among Dubai population

Characteristics	Prevalence %			^*P* value
	Normotension	PHTN	HTN	
*Self-reported chronic disease*				
No chronic diseases	41.36	31.70	26.94	< 0.001
Have chronic diseases	13.82	17.36	68.82	
*History of high cholesterol*				
No cholesterol	38.61	30.46	30.93	< 0.001
Have cholesterol	32.37	25.82	41.81	
*Have diabetes*^#^				
No diabetes	42.64	32.55	24.81	< 0.001
Have diabetes	19.20	19.42	61.38	
*History of chest pain*				
No	37.89	30.23	31.88	< 0.001
Yes	32.21	16.16	51.63	
*BMI*				
Normal	51.18	23.90	24.92	< 0.001
Overweight	37.29	25.23	37.48	
Obese	30.43	13.75	55.82	

BMI: Body Mass Index

*HTN* hypertension, *PHTN* prehypertension

^^^χ^2^ test

^#^Diabetes here is diagnosed both by tests and the self-reported cases

The associations between behavioral risk factors and PHTN and HTN were shown in Table 3. Using univariate analysis, the current findings reflected that tobacco use, smoking status, passive smoking, eating adequate fruits and vegetable consumption, alcohol consumptions, being physically active, all showed significant statistical associations with the prevalence of PHTN and HTN among the Dubai population at the level of *P* < 0.001. Multivariate logistic regression analysis showed that occupations and being overweight were significantly associated with PHTN (compared to normotensive), as shown in Table 4. Age groups and gender were not significantly associated with PHTN. In terms of the current occupation of the participants, skilled and services workers had a higher risk of PHTN, compared to the reference group (professionals) [OR 4.28 (95% CI 1.80–10.17), OR 2.12 (95% CI 1.13–4.02), respectively]. Individuals with a status of overweight associated with a higher prevalence of PHTN compared to the normal weighted [OR 2.01 (95% CI 1.03–3.92)], while obesity was not associated with PHTN. Correlates of HTN as compared to normotensive through multivariable logistic regression analysis were also shown in Table 4. Compared to the reference group (18–29 years), only people aged 50–59 years and those 60+ years had a greater correlation of developing HTN [OR 3.35 (95% CI 1.04–10.85), OR 7.59 (95% CI 7.13–8.07), respectively]. Males had a higher risk of HTN than females [OR 5.02 (95% CI 1.61–15.64)]. Similar to PHTN, those who work in skilled and service works had a 4.62 times higher risk of HTN (95% CI 2.04–10.47), compared to the reference group (professionals). Being overweight carries 2.56 times the risk for HTN compared to normal-weight subjects (95% CI 1.32–5.00). In addition, obese subjects had a 5.47 times greater correlation of HTN compared to the same reference group (95% CI 2.57–11.64). Neither smoking nor alcohol drinking was correlated with the risk of developing HTN in the present sample. On the other hand, physically active individuals were less likely to develop HTN [OR 0.51 (95% CI 0.29–0.92)], as expected.

**Table 3:** Behavioral risk factors associated with PHTN and HTN among Dubai population

Characteristics	Prevalence %			^^^*P* value
	Normotension	PHTN	HTN	
*Have used tobacco*				
No	38.28	30.41	31.31	< 0.001
Yes	35.40	27.31	37.29	
*Current smoking status*				
No	42.58	15.97	41.45	< 0.001
Yes	34.51	28.71	36.78	
*Exposure to passive smoking*				
No	36.72	30.20	33.08	< 0.001
Yes	44.22	27.06	28.72	
*Adequate fruits and vegetables consumption*				
No	38.84	29.24	31.92	< 0.001
Yes	35.31	30.94	33.75	
*Drinking alcohol*				
No	38.45	30.37	31.19	< 0.001
Yes	34.48	27.63	37.89	
*Physically active*				
No	37.51	29.59	32.90	0.000
Yes	13.53	31.88	54.59	

*HTN* hypertension, *PHTN* prehypertension

^χ^2^ test

**Table 4:** Logistic regression analysis for correlates of PHTN compared to normotensive and HTN compared to normotensive*

Characteristics	PHTN		HTN	
	OR (95% CI)	*P* value	OR (95% CI)	*P* value
*Age group (Years)*				
18–29	Reference group		Reference group	
30–39	1.34 (0.67–2.72)	0.40	2.11 (0.98–4.27)	0.30
40–49	1.28 (0.58–2.85)	0.53	1.51 (0.69–3.35)	0.08
50–59	2.33 (0.68–7.99)	0.17	3.35 (1.04–10.85)	< 0.05
60+	2.33 (0.34–2.87)	0.34	7.59 (7.13–8.07)	< 0.001
*Gender*				
Female	Reference group		Reference group	
Male	1.72 (0.71- 4.18)	0.22	5.02 (1.61- 15.64)	< 0.001
*Occupation*				
Professionals	Reference group		Reference group	
Skilled and service workers	4.28 (1.80–10.17)	< 0.001	4.62 (2.04–10.47)	< 0.001
Elementary/unskilled workers	2.12 (1.13–4.02)	< 0.05	1.27 (0.70–2.33)	0.430
^*BMI*				
Normal	Reference group		Reference group	
Overweight	2.01 (1.03–3.92)	< 0.05	2.56 (1.32–5.00)	< 0.001
Obese	2.06 (0.90–4.69)	> 0.05	5.47 (2.57–11.64)	< 0.001
*Smoking status*				
No	Reference group		Reference group	
Yes	1.05 (0.45–2.48)	0.89	1.07 (0.49–2.42)	0.850
*Alcohol consumption*				
No	Reference group		Reference group	
Yes	0.99 (0.53–1.88)	0.99	1.71 (0.96–3.06)	0.700
*Physically active*				
No	Reference group		Reference group	
Yes	0.97 (0.55–1.74)	0.93	0.51 (0.29–0.92)	< 0.050

^BMI, Body Mass Index; OR, odds ratio; CI, Confidence Interval at 95%; *P *value < 0.05; HTN, Hypertension; PHTN, Prehypertension

^*^The reference category is normotensive

^#^Parameter is set to zero because it is redundant

## Discussion

### Prevalence of PHTN and HTN

The current findings highlighted a significant burden of both PHTN and HTN in the studied population. The present findings showed that the prevalence of PHTN was 29.8%, which was similar to the figures reported elsewhere [14, 16, 22–24]. However, this result was lower than the figures reported in the neighboring Arabian Gulf countries (37.4%, 45%, and 54.9% in Bahrain, Oman, and Saudi Arabia, respectively) [25–27]. Regarding the prevalence of HTN, the present data also revealed that it affects about one-third (32.5%) of adults in Dubai. This is comparable to the results from other countries worldwide [12, 28–30]. Our prevalence lies within the range of HTN reported in the Gulf States (26.1%, 34%, and 37% in Saudi Arabia, Oman, and Kuwait, respectively) [26, 31, 32]. The differences observed in the prevalence between the Dubai population and other studies with respect to PHTN and HTN could be attributed to dietary and lifestyle factors, cultural and social differences, and the adopted research methodologies. It is worth mentioning that, the proportion of adults with HTN in Dubai was higher than the national average for the UAE of 28.8% [33]. This might be due to the unique demographics and different dynamics of the population of Dubai, compared to the other Emirates [34].

### Risk factors associated with PHTN and HTN

In the current survey, the logistic regression analysis showed that age, gender, occupation, and BMI were significantly associated with the risk of having HTN. However, physical activity was shown as a protective factor. At the same time, occupation and BMI were the only associated factors with PHTN. The current findings showed that advanced age was associated with an increased risk of HTN. Almost all the studies carried out in different populations had shown a somehow consistent increase in BP with aging [22–29]. Vascular abnormalities might contribute to the high prevalence of HTN in the elderly [35].

Data from the survey revealed a huge gender gap in HTN prevalence between males and females in Dubai. Males were found to be five times more likely to be hypertensive than females. Other studies have confirmed these findings [22–27, 32]. The gender differences in hypertension are mainly due to biological factors including sex hormones that keep lower levels of HTN in women, among other factors [36–38]. Behavioral risk factors, including high BMI, smoking, alcohol consumption, and physical inactivity, differ in complex ways between males and females as they play some protective effects in women [28, 36–38]. In addition, women utilize healthcare services more frequently which could have a positive effect on controlling diseases like HTN [29, 36].

The present study showed that being overweight was associated with both PHTN and HTN. However, obesity was proven only as a risk factor for HTN within our population. This can be explained by the fact that obese individuals could progress quickly to the actual HTN from the PHTN status [39]. It is worth noting that, 62.1% of adults in Dubai are either overweight or obese (41.3%, 20.8%, respectively) [17]. International studies revealed a direct association between increased BMI and HBP and identified obesity as a major risk factor for HTN and PHTN [25–30]. The mechanisms by which obesity contributes to the development of HTN including insulin resistance among other pathophysiological changes [40, 41].

Compared to professionals, skilled and service workers endured a higher risk for both PHTN and HTN within the current population. The influence of occupation as a predictor for high BP has rarely been stated in other studies [28, 32, 42]. Given that there are some variations in the definitions of the occupational categories among different studies, contradictory findings have been reported regarding this association [14, 43]. Similar to the present results, service occupations had a significantly increased prevalence of HTN [44, 45]. However, other studies found that unskilled workers were found to have more HTN than professionals and managerial ones [45, 46]. The difference in job strains, work environment, and levels of decision-making are among the factors that underlie the differences in HTN prevalence by occupations [47].

In the current study, physical activity was significantly inversely associated with the risk of developing HTN. Consistent evidence from research has endorsed the favorable effects of exercise and physical activities on the control of BP and prevention of HTN [14, 15, 48, 49]. Still, there are many unaddressed questions in the nature of the association between physical activity and prevention of HTN in terms of the types, levels and duration of physical activity needed, particularly in high-risk individuals [48].

Unexpectedly, the current findings showed that smoking and alcohol drinking was not associated with PHTN and HTN within the population of Dubai. Our results were not in agreement with other studies [14, 27, 50], since some existing evidence shows that smoking and alcohol drinking were among the risk factors for high BP. The discrepancy between our results and other studies might be explained by the existence of a social stigma attached to smoking and alcohol drinking, especially among females in the Arab region. Therefore, smoking and alcohol drinking could be underreported among the Dubai population [51].

This study has few limitations. First, the survey used data derived from both self-reports and physical measurements. The information collected on a self-reported basis is subjected to some form of bias. Subjectivity, underwriting, or misreporting associated with self-reporting cannot be ignored. A second limitation is that the survey did not collect some important variables such as the family history of HTN, salt and dietary intake of the studied population. Finally, as this survey used a cross-sectional design, the cause and effect relationship could not be established. Despite the above limitations, the strength of this study lies in that it is based on a population survey, which allows a representative population sample. In addition, the current study is the first study to shed light on the prevalence of PHTN and HTN and their correlates at the population level in the emirate of Dubai.

## Conclusions

This is the first study to look at PHTN and HTN among adults in Dubai. The current findings demonstrated that both PHTN and HTN are prevalent among Dubai adults. Clearly, males are at higher risk of having HTN than females. Advanced age is identified to be an independent risk factor for both PHTN and HTN. Further findings showed that overweight, obesity, and occupations significantly predicted the odds of HTN. On the other hand, physical activity was a protective factor from HTN. Therefore, to decrease the burden of PHTN and HTN, early lifestyle modifications including reducing behavioral risk factors might be necessary to prevent the conversion from PHTN to HTN. This can be achieved through improvements in the screening measures and the application of community-based screening services for the early detection of PHTN as well as HTN. Developing and applying a screening tool, such as the cardiovascular risk score, at the primary health care level to detect the high-risk population is recommended. Programs directed at raising the awareness of the community towards the prevention strategies and the health implications of HTN and PHTN are necessary. Developing community initiatives/programs such as food labeling, nutrition interventions, and physical activity programs could be considered as good measures towards the control of BP and its associated cardiovascular risk reduction. Multispectral stakeholder’s engagement in these programs at different levels is essential. Well-designed future studies are needed to assess the direct association between the different occupation styles, dietary patterns, and types and patterns of physical activity, and the BP status.

## Data Availability

The data that support the findings of this study are from the Dubai Health Authority. However, restrictions apply to the availability of these data; thus, these data are not publicly available. Data are available from the corresponding author upon responsible request with permission from the Dubai Health Authority.

## Abbreviations

BMI: Body Mass Index
BP: Blood pressure
CI: Confidence intervals
DHA: Dubai Health Authority
DHHS: The Dubai Household Health Survey
DBP: Diastolic blood pressure
HTN: Hypertension
OR: Odds ratios
PHTN: Prehypertension
SBP: Systolic blood pressure
WHO: World Health Organization
